# LncRNA–Protein Interactions: A Key to Deciphering LncRNA Mechanisms

**DOI:** 10.3390/biom15060881

**Published:** 2025-06-17

**Authors:** Zuoneng Wang, Muhammad Aftab, Zigang Dong, Yanan Jiang, Kangdong Liu

**Affiliations:** 1 Tianjian Laboratory of Advanced Biomedical Sciences, Academy of Medical Sciences, Zhengzhou University, Zhengzhou 450001, China; 2 State Key Laboratory of Esophageal Cancer Prevention and Treatment, Zhengzhou 450000, China; 3 Department of Pathology and Pathophysiology, School of Basic Medical Sciences, Zhengzhou University, Zhengzhou 450001, China; 4 China-US (Henan) Hormel Cancer Institute, Zhengzhou 450008, China

**Keywords:** long non-coding RNA, RNA-binding proteins, lncRNA-protein interactions, structure-function relationship, therapeutic targets

## Abstract

Long non-coding RNAs (lncRNAs) have emerged as pivotal regulators in a multitude of biological processes. However, their functional basis, particularly structure-based functional characteristics, remains elusive. lncRNAs exert their influence primarily through intricate interactions with various cellular components. Among these, interactions with proteins have garnered increasing attention. Recent research highlights the significance of the interactions with proteins as a plausible mechanism underlying lncRNA functions. Here, we delve into the interactions between lncRNAs and RNA-binding proteins (RBPs), explore their implications in cellular processes, and examine bioinformatic and experimental approaches for characterizing these interactions. We introduce an innovative ISD strategy to decipher the mysterious mechanism of lncRNAs. Through reviewing the recent advances in the study of proteins and their complexes, we incorporate the ISD strategy into our integrated structural analysis pipeline for comprehensively understanding the structure-function relationship of lncRNAs. Advances in the development of innovative therapeutic approaches based on lncRNA-protein interactions (LPIs) are reviewed accordingly.

## 1. Introduction

LncRNAs (Long non-coding RNAs) are non-coding RNAs that harbor sequences over 200 base pairs [1]. Although present at a low abundance, an increasing number of studies indicates that lncRNAs play vital roles in various cellular processes, such as differentiation and signal transduction [2]. Due to their lack of protein-coding capability, lncRNA sequences typically provide limited insights into their functional roles. Predicting lncRNA function solely from primary sequence similarity is also challenging, as their molecules exhibit low conservation across species [3,4]. A significant portion of lncRNAs adopts two-dimensional (2D) or three-dimensional (3D) structures when interacting with partner molecules to carry out cellular functions [5]. Even a combination of multiple modules, formed by conserved patches within the sequence, can be defined as functional motifs. However, determining the structures of such motifs, as well as the entire lncRNA molecule, remains a significant challenge [6]. Non-conserved primary sequence fragments are often active and tend to be flexible, which contributes to the high heterogeneity of RNA molecules in structural study pipelines. Additionally, the low abundance of lncRNAs and the presence of multiple distinct modules necessitate efficient approaches to obtain properly folded RNAs with stable structures [7,8].

The functionalization of most lncRNAs involves direct interaction with various partners. Notably, the protein partner has been drawing more and more attention due to its significant roles in LncRNA function [9]. Therefore, proteins are confirmed to be the first and principal partner of lncRNAs [10,11]. The multiple modular scaffolds within lncRNA sequences provide suitable binding interfaces or docking pockets, assembling diverse combinations of proteins [12]. For instance, lncRNAs can function as components of ribonucleoprotein complexes (RNPs) to support molecular functions. LncRNA-protein interactions (LPIs) are accepted as major functional units in metabolic processes, many of which have been shown to be closely related to human diseases [13,14,15,16,17]. Accordingly, the development of recently emerging and progressively improving methods to explore LPIs has greatly advanced our understanding of lncRNA mechanisms.

To identify LPIs, the mainstream strategy is to isolate their interactome based on the pulldown principle, followed by characterization of these interactions. For instance, workflows can be designed around a specific lncRNA to systematically explore its binding proteins [18]. An alternative strategy involves separately centering investigations on the protein partners, with the two strategies ultimately combined for complementary validation of their interactions. Recent molecular docking techniques, combined with various algorithms, allow for the investigation of LPIs through computer simulations. Commonly used web servers for such studies include, but are not limited to, HADDOCK and P3DOCK [19,20]. The accuracy of docking predictions can be greatly enhanced when structural information about the protein or lncRNA is available, and these structures can subsequently be experimentally validated. In alignment, various databases such as RBP2GO, RNA Bricks, and NPIDB cataloging LPI networks are emerging and undergoing continuous refinement [21,22,23]. Some of these now not only provide interaction details but also include information on specific binding motifs of lncRNA. To optimize the use of these resources, guide matrices and advanced search functionalities have been developed, allowing for more precise retrieval of relevant data [24]. In recent years, machine learning-based algorithms for predicting LPIs have been gradually maturing and showing great potential. Particularly deep learning methods, also known as artificial neural networks, have been shown to achieve high accuracy in predicting lncRNA-binding proteins. For instance, the DRPScore approach has been reported to evaluate native-like structures with a success rate of 91.67% on a testing set of RNA-protein complexes [25].

Overall, identifying or predicting the proteins that interact with lncRNAs cannot fully capture the molecular mechanisms underlying the structure and function of lncRNA. particularly the 3D structural mechanisms that are crucial for understanding the functional basis of lncRNAs and discovering disease prevention strategies. Recent research has provided us with new insights: Once lncRNA-binding proteins are identified, these proteins can, to some extent, be considered stabilizers of the lncRNA [26]. By applying a broader range of existing protein- or RNA-based approaches, it is becoming feasible to elucidate structural mechanisms of lncRNAs from the perspective of their protein partners or to consider lncRNA-protein complexes as independent operational units in cellular processes when investigating lncRNA’s roles in human diseases.

Therefore, in the first part of this review, we outline recent advances in uncovering the cellular mechanisms of lncRNAs, primarily through their protein-binding partners, including the biological consequences of lncRNA–protein interactions in cellular regulation and human disease. In the following core sections, we focus on experimental approaches for studying LPIs, from current methods for identification of LPIs to the structural–functional analysis of lncRNAs in their protein-bound state. We provide an in-depth discussion on the determination of secondary and tertiary structures and the study of structure–function relationships of lncRNAs within these complexes. We carefully examine emerging approaches in this area, attempt to formulate a systematic approach for overcoming the limitations mentioned above, and propose strategies to advance future research. We highlight our ISD strategy as a productive approach for uncovering the multiple roles of lncRNAs, particularly when integrated into our structural analysis framework. Naturally, the rising recognition of LPIs in diseases, along with ongoing efforts to develop therapies targeting these interactions, underscores the significance of lncRNAs in the body, which we further discuss in the final section.

## 2. Mechanisms of LncRNA–Protein Interactions

LncRNAs exhibit remarkable functional diversity through their ability to interact with one or more protein partners via a range of molecular mechanisms. Although lncRNAs are generally more abundant in the nucleus, they are also more prevalent in the cytoplasm than previously thought. This difference in subcellular localization has important implications for the functional roles of lncRNAs in various biological processes [27].

**Figure 1:**
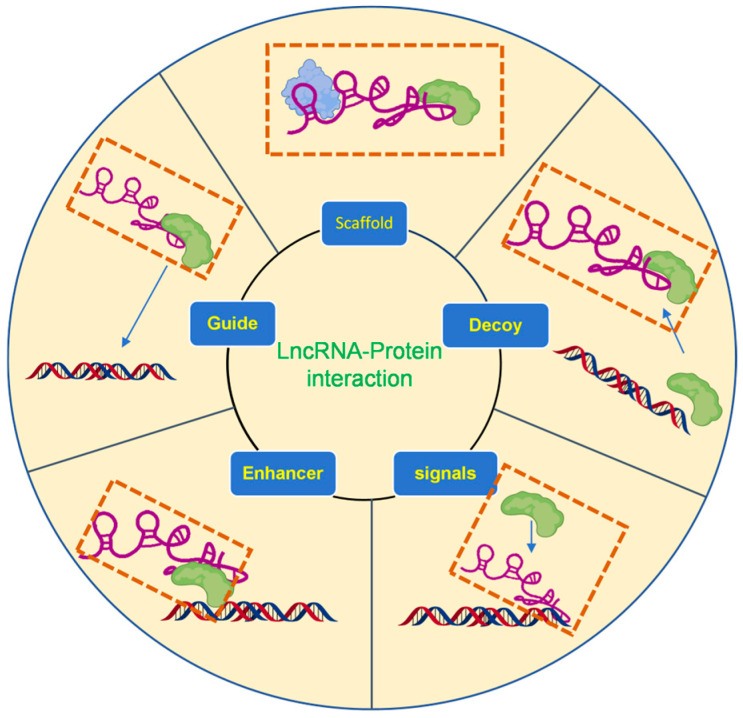
Schematic illustration of major archetypes of LPIs (dashed boxes).

Early studies categorized lncRNAs into five archetypal functional classes: guides, scaffolds, decoys, enhancers, and signals, each representing a distinct mode of action across various biological processes [28,29]. These archetypes remain highly relevant when examining lncRNA–protein interactions, as each type tends to involve specific modes of protein binding and regulatory mechanisms. For instance, guide lncRNAs recruit chromatin-modifying or transcriptional regulatory proteins to specific genomic loci, thereby directing site-specific gene regulation. Scaffold lncRNAs serve as structural platforms that bring together multiple proteins into functional complexes, often facilitating enzymatic cascades or chromatin remodeling. Decoy lncRNAs bind and sequester transcription factors or RNA-binding proteins (RBPs), preventing them from acting on their usual targets and modulating downstream signaling. Enhancer-like lncRNAs interact with transcriptional co-activators or mediators, helping to stabilize or spatially organize protein assemblies that activate gene expression. lncRNAs can also function as signals by integrating spatial and temporal expression patterns to mediate physiologically specific recruitment of protein partners [30], as Figure 1 shows.

Importantly, these categories are not mutually exclusive. A single lncRNA may contain multiple structural or sequence domains that mediate interactions with different proteins either simultaneously or under different conditions. This modular and functionally dynamic architecture allows lncRNAs to act pleiotropically, playing distinct regulatory roles depending on the biological setting. The availability of protein partners, subcellular localization, and the lncRNA’s structural configuration collectively determine the functional output of these interactions [31]. This classification enhances our understanding of how lncRNAs interact with protein partners and contribute to a broad spectrum of cellular functions.

## 3. Biological Consequences of LPIs

### 3.1. Subcellular Localization and Functional Compartmentalization

Sequence-encoded signals are believed to guide lncRNA localization, yet their low sequence conservation and extensive alternative splicing complicate the prediction of subcellular distribution [32]. lncRNAs also exhibit pronounced tissue-specific expression, and their localization is often influenced by the availability of local protein partners, highlighting a cooperative interplay between lncRNAs and proteins in defining both spatial positioning and compartment-specific activity [33,34]. Interactions with RBPs help determine whether lncRNAs are retained in the nucleus or exported to the cytoplasm, especially when functional data is limited [35]. Chromatin-associated lncRNAs are typically found in the nucleus, participating in transcriptional regulation through chromatin remodeling [36]. However, recent evidence shows that some of these lncRNAs also localize to the cytoplasm, where they scaffold signaling protein complexes such as TLR–TRIF [37]. LncRNAs like LINC00473 and SAMMON are found at specific subcellular sites, including mitochondria and lipid droplet interfaces, via direct protein interactions. Some others exhibit dynamic localization patterns influenced by physiological conditions [38]. Given that classification of lncRNAs is often based on subcellular localization, understanding protein-mediated compartmentalization provides important clues to their biological roles. For example, in lung adenocarcinoma, reduced LINC00472 expression is closely linked to clinical outcomes, with its function dependent on interactions with proteins such as YBX1 [39].

Owing to their versatile modes of protein interaction, lncRNAs frequently act as structural and regulatory hubs that assemble protein complexes into distinct functional compartments within the cell. For example, GlycoLINC orchestrates glycolytic metabolism by bringing together key enzymes into a multi-component complex [40], while SLERT regulates RNA polymerase I activity via stoichiometric interaction with DDX21 [41]. In certain conditions, multiple lncRNAs co-localize with shared protein partners to form higher-order regulatory structures, such as the nuclear bodies in Prader–Willi syndrome, where sno-lncRNAs and SPA-lncRNAs recruit splicing regulators, including RBFOX2 and TDP43, through sequence-specific motifs [42,43,44]. lncRNAs also contribute to the formation of membraneless and phase-separated condensates. For example, NORAD sequesters PUM proteins within such condensates, modulating RBPs’ availability and function.

### 3.2. Regulation of Gene Expression

LncRNAs regulate gene expression through interactions with protein partners, ranging from influencing DNA repair, chromatin remodeling, and transcription [45,46].

For example, GUARDIN, a p53-inducible lncRNA, binds to BRCA1 and BARD1, facilitating the recruitment of DNA double-strand break (DSB) repair complexes [13]. Similarly, NORAD interacts with RBMX and TOP1 to form a ribonucleoprotein complex that maintains mitotic integrity and prevents chromosomal instability [47]. Multiple other lncRNAs, such as LINP1, NIHCOLE, and LRIK, enhance non-homologous end joining (NHEJ) by scaffolding Ku70/Ku80 and recruiting DNA-PKcs to DSB sites [48]. lncRNAs also regulate chromatin states and gene transcription. Some act by modifying chromatin architecture, such as L1 elements, which interact with EZH2 and SUZ12 to deposit H3K27me3 marks and regulate cortical development [49]. Others exert dual regulatory effects: HOTAIR activates GLI2 transcription via interaction with the androgen receptor (AR), while ZFAT-AS1 suppresses CDX2 by recruiting PRC2 to its promoter, leading to gene silencing through H3K27me3 [50,51].

Epigenetic gene regulation by lncRNAs frequently involves DNA methylation machinery and chromatin modifiers. For instance, CDKN2B-AS1 can form RNA–DNA triplexes to recruit proteins like EZH2 and CTCF, influencing local methylation status. The nuclear pore protein NUP153 represents another layer of lncRNA-mediated chromatin regulation. NUP153 modulates gene activation by coordinating CTCF and cohesin occupancy at topologically associating domain (TAD) boundaries, influencing bivalent gene expression in embryonic stem cells [52]. Similarly, Meg3 facilitates TAD formation at the Dlk1-Dio3 locus by binding CTCF, thus enabling cis-silencing through 3D chromatin architecture remodeling [53]. During epidermal differentiation, NEAT1 exemplifies lncRNA-mediated transcriptional control. Initially repressed by ΔNp63-HDAC1, NEAT1 levels rise upon differentiation, forming paraspeckles and associating with promoters of epithelial transcription factors to sustain their expression [54].

### 3.3. Modulation of Cellular Processes

LPIs influence a wide array of cellular functions by shaping the expression and activity of key regulatory molecules. These complexes contribute to essential processes such as cell proliferation, immune regulation, and metabolism in various signaling pathways.

For instance, SDNOR promotes granulosa cell proliferation and resistance to oxidative stress by inhibiting SOX9, a key factor in steroidogenesis and cell cycle regulation [55], while CHASERR enhances glioma progression by upregulating TRIM14 through miR-6893-3p sequestration and activating the PTEN/p-Akt/mTOR and Wnt/β-catenin pathways [56].

In muscle development, LncMREF cooperates with the chromatin remodeler Smarca5 to enhance chromatin accessibility and activate MyoD expression via recruitment of p300/CBP and H3K27ac deposition [57], while Has2os promotes myocyte fusion and differentiation by inhibiting the JNK/MAPK pathway [58]. Similarly, SNHG1 enhances STAT3 phosphorylation, promoting chondrogenic differentiation and angiogenesis in jaw bone marrow mesenchymal stem cells [59]. In tumor settings, lncRNAs reshape the immune landscape by modulating macrophage plasticity, T cell activity, and MDSC function, contributing to immune evasion and therapy resistance [60]. In hepatocellular carcinoma, MIAT promotes immune escape by stabilizing STAT3 through miR-411-5p inhibition, resulting in increased PD-L1 expression [61]. Integrative studies have also revealed immune-related lncRNA–protein networks across diseases like pulpitis, where distinct molecular subtypes have been characterized [62]. Moreover, LPIs contribute to metabolic regulation by organizing enzymatic complexes, as exemplified by glycoLINC, which scaffolds glycolytic enzymes to coordinate metabolic flux [6,63].

### 3.4. Implications in Human Diseases

**Figure 2:**
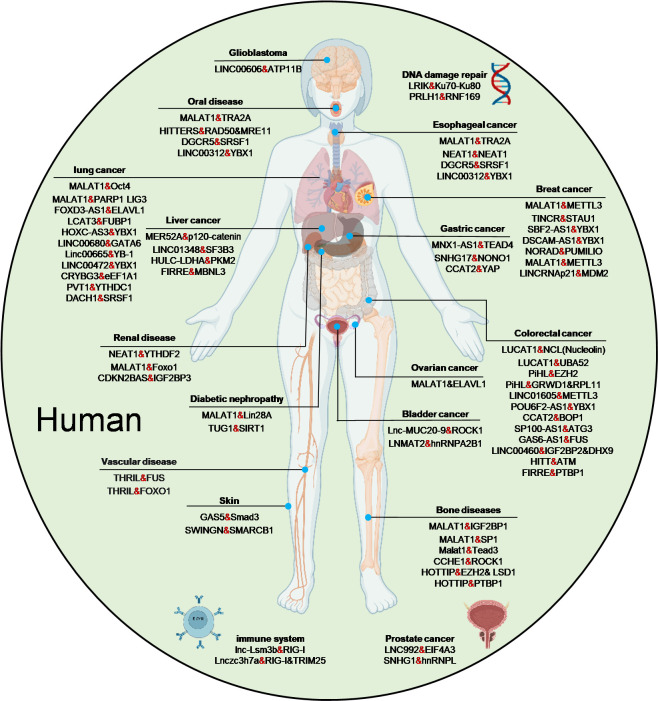
Recent uncovered lncRNA-protein partners and their associated diseases [39,64,70,71,72,73,74,75,76,77,78,79,80,81,82,83,84,85,86,87,88,89,90,91,92,93,94,95,96,97,98,99,100,101,102,103,104,105,106,107,108,109,110,111,112,113,114,115,116,117,118,119,120,121,122,123,124,125,126,127,128,129,130,131,132,133,134,135,136,,137,138,139,140,141,142,143,144,145,146,147,148,149].

The biological consequences of LPIs are increasingly recognized in diverse diseases. For instance, LINC00472 is significantly downregulated in lung adenocarcinoma, correlating with poor prognosis and TNM stage [38]. MALAT1 aggravates renal tubular injury in diabetic nephropathy by enhancing AMPK/mTOR signaling through stabilization of the LIN28A–Nox4 interaction [64]. In Prader–Willi syndrome, the loss of sno-lncRNAs and SPA-lncRNAs from chromosome 15q11–13 disrupts nuclear body formation by impairing interactions with splicing factors such as RBFOX2 and TDP43 [42,43]. A substantial number of LPIs are implicated in cellular signaling pathways, and many have been experimentally validated as contributors to disease progression. These data are documented in comprehensive resources like RAID, NONCODE, and NPInter [65,66,67], while specialized databases such as Lnc2Cancer and LncPCD provide focused insights into LPIs involved in cancer and programmed cell death [68,69]. Figure 2 summarizes recent findings on how LPIs are implicated in disease processes.

## 4. Experimental Approaches for Studying LPIs

### 4.1. Identification of LPIs

**Figure 3:**
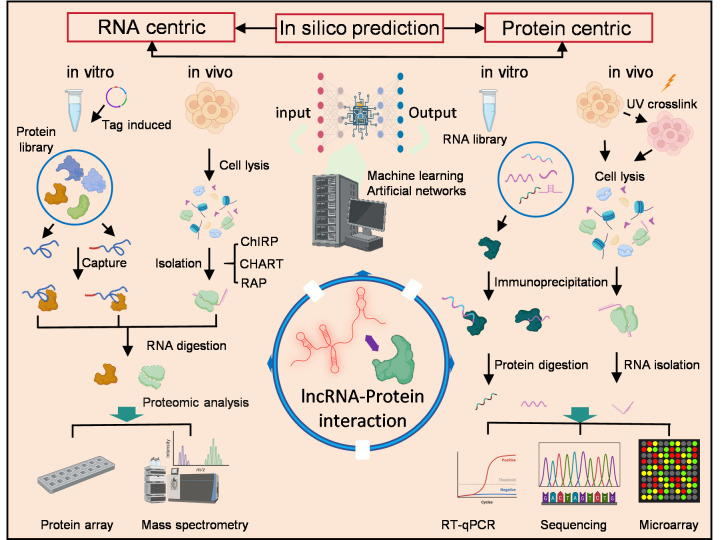
Schematic description of current approaches for detecting LPI.

A variety of technologies that focus on either proteins or lncRNAs have been developed to identify LPIs nowadays [150]. Experimental methods generally rely on the pull-down principle and fall into categories of protein-centric and RNA-centric approaches, as Figure 3 shows.

In protein-centric techniques, the target protein is usually isolated at an early stage, followed by sequencing of associated lncRNA to map the interactions along the transcriptome. In the case of in vitro performances, the protein of interest is immobilized on a matrix, and a synthetic lncRNA library flows through the bound protein. High-affinity lncRNAs (Hits) are pulled down and subjected to sequencing. Advanced representatives are RNA Bind-n-Seq (RBNS) methods, which allow statistical modeling and the determination of dissociation constants between RNA and its binding proteins by deep sequencing [151,152]. A limitation of this method is that it is difficult to distinguish false positives. LncRNA hits that do not bind a protein inherently may show certain affinity and get selected, particularly when selection cycles are not conducted under stringent conditions. To mitigate this, affinity thresholds should be adjusted to reflect natural ligand interactions, and RNA libraries should be generated from cellular transcriptomes or by utilizing genomic fragments [153].

Alternatively, in vivo approaches have gained popularity in recent years. In vivo protein-centric methods usually utilize immunoprecipitation (IP) to isolate RNA-protein complexes, followed by co-purified RNA detection using microarrays (RIP-chip, RNA Immunoprecipitation-Chip) or RNA immunoprecipitation sequencing (RIPSeq) [154,155]. While these methods identify bound transcripts, they do not provide direct information about the binding site. Advanced methods were developed to recognize the binding sites and screen bound complexes in parallel with high throughput. For example, crosslinking immunoprecipitation RNA-sequencing (CLIP-Seq) and some of its variants, including HITS-CLIP, PAR-CLIP, and iCLIP [156,157]. These methods take advantage of covalent bonds induced by ultraviolet (UV) light between RNA and protein at the binding site, enabling the isolation of protein–RNA complexes by immunoprecipitation at high accuracy. The bound RNA is subsequently reverse-transcribed for next-generation sequencing. Crosslinking protein to modified lncRNA facilitates precise identification of protein types and their binding sites within the lncRNA sequence [158,159].

To enhance specificity and reproducibility, enhanced CLIP (eCLIP) incorporates an input control and has become a standardized technique within the ENCODE [160]. In addition, formaldehyde crosslinking immunoprecipitation (fCLIP) uses formaldehyde instead of UV to capture interactions that are otherwise difficult to crosslink, expanding the detectable spectrum of RNA–protein complexes [161]. Given the sequencing-based nature of these techniques, appropriate computational tools such as HTSeq-CLIP are essential for accurate data interpretation and downstream analysis [162].

To detect novel RBPs that interact with specific lncRNAs, an RNA-centric approach is commonly used instead (Figure 3). In this technique, the RNA of interest is purified and used as bait to capture interacting proteins. The associated protein complexes are then identified by enzymatically digesting the RNA and analyzing the proteins using mass spectrometry (MS), protein arrays, or other techniques [163,164]. In vitro-transcribed (IVT) RNAs tagged with biotin can be captured using streptavidin-conjugated beads, facilitating the isolation of the interacting protein network [165].

Due to the fact that synthetic RNA may misfold, leading to nonspecific binding with proteins in cell lysis solution. An in vivo RNA-centric method was developed to avoid this problem and preserve the context of natural RNA-protein interactions [166,167,168]. For example, RNA purification-based chromatin isolation (ChIRP), capture hybridization analysis of RNA targets (CHART), and RNA antisense purification (RAP) all isolate the relevant endogenous lncRNA using antisense oligonucleotides [169,170,171,172]. Ultimately, proteins that are eluted undergo analysis via mass spectrometry (MS). Certain modifications were also employed to explore protein with a specific RNA. For example, Pant et al. reported a method called MORPH-MS (Multiple Oligo-assisted RNA Pulldown via Hybridization) that uses a biotinylated oligo to collect all the antisense oligos rather than several separate biotinylated oligos. This method effectively extracts the relevant RNA along with its associated proteins [173]. One limitation of these methods is that their performance often depends on the efficiency of oligo hybridization and the abundance of target transcripts, making optimization crucial for low-expression lncRNAs. More recently, proximity labeling-based RNA-centric approaches such as RPL and CARPID have expanded the landscape of detectable LPIs in live cells. These techniques rely on catalytically inactive Cas13 (dCas13) fused to enzymes like APEX2 or biotin ligase, enabling biotinylation of proteins in close proximity to a target lncRNA. This allows comprehensive proteomic profiling of lncRNA-associated proteins under native cellular conditions [174,175].

In addition to experimental methods, in silico methods such as artificial neural networks have been developed to predict LPIs. These include integrated machine learning, combined graph auto-encoders (LPICGAE), deep autoencoder & marginal fisher analysis (DFRPI), and DeepLPI [176,177,178,179]. Further examples of similar techniques can be found in articles [180,181,182,183]. Moreover, online tools have been developed and are available for inputting predictions directly, for example, RNAincoder, SoCube, Nucpred, DeepBtoD, DeepA-RBPBS, and PST-PRNA [184,185,186,187,188,189]. In addition, databases such as POSTAR3 integrate large-scale CLIP-seq datasets with functional annotations to systematically analyze RBP binding sites, RNA structural features, disease-associated mutations, and miRNA-mediated decay events, thereby offering a multi-dimensional reference for CLIP data interpretation [190].

Compared with experimental approaches, in silico tools offer high-throughput screening capabilities and require no physical material, making them suitable for prioritizing candidate LPIs. However, prediction quality heavily depends on the training data and algorithmic features. Thus, experimental validation remains essential, particularly when moving toward functional interpretation. Table 1 illustrates the characteristics of common methods for detecting LPIs.

### 4.2. Structural Insights into LncRNA–Protein Binding

Most lncRNAs possess higher-order structures that are often critical for their function. Various methods have been developed to study the structure of lncRNAs. Notably, some studies not only address the structural prediction challenge but also explore its application in RNA-based therapeutic development. In the case of HOTAIR, which promotes EMT by enabling Snail-mediated EZH2 recruitment, a deletion mutant (HOTAIR-sbid) retaining the Snail-binding domain but lacking the EZH2-binding region acted as a dominant negative. HOTAIR-sbid impaired tumor cell invasiveness, motility, and TGFβ-induced EMT, highlighting how domain-based strategies can circumvent structural challenges in lncRNA-targeted therapy [204]. From functional analysis, the roles of lncRNAs can be inferred even without full structural resolution. For example, chemical probing of lncTCF7, a conserved lncRNA implicated in cancer, revealed modular structural domains and conserved regions, one of which overlaps with a known protein-binding site. As the first structural model of lncTCF7, this work highlights how domain-level insights can guide functional understanding in the absence of complete structural information [205]. We will further discuss the methods used to study lncRNA structure in the following sections.

#### 4.2.1. Probing LncRNA Secondary Structure

In lncRNA molecules, complementary base pairs typically form structures such as bulges, junctions, loops, helices, subdomains, and pseudoknots, which are defined as secondary structures [206]. In many cases, the function of lncRNA is affected, and to some extent, determined by their highly conserved secondary structures. Especially when they provide recognition sites for unique protein components. Therefore, the knowledge of secondary structure is a primary and essential information source for the identity of protein interplay [207,208]. For instance, NORAD interacts with PUMILIO proteins to preserve genomic stability. An analysis of its sequence identified multiple repeated secondary domains (NRUs). Each domain features PUMILIO response elements (PRE) that exhibit strong and specific binding to PUMILIO proteins (PUM1 and PUM2) [209,210].

Conventional probe methods primarily rely on thermodynamic calculations and dynamic programming. Such as in vitro chemical probing, which has produced extremely accurate secondary structures that can also be confirmed by X-ray crystallography [211]. It’s worth noting that a single sequence of RNA (e.g., riboswitch RNA) may form multiple and mutually competitive secondary structures to determine certain functions. In this context, the binding ligand concentration causes a change in the secondary structure’s equilibrium, which either creates or destroys an ON/OFF transcriptional terminator state [212]. Meanwhile, certain RNA can dynamically adjust secondary structures in response to temperature changes [213]. The development of high-throughput sequencing (HTS) technologies has revolutionized RNA structure analysis, enabling the simultaneous examination of thousands of RNA molecules or entire transcriptomes. Additionally, the RNA-protein interaction interface can be indirectly probed by observing changes when comparing free lncRNA with RNA-bound states. These advancements have greatly enhanced our understanding of RNA structure [214,215]. Recently, a method called LLPS-CLIR-MS (liquid-liquid phase separation combined with cross-linking of isotope-labeled RNA and tandem mass spectrometry) was reported to investigate protein-RNA interactions inside biomolecular condensates. This technique enables the characterization of RNA-protein interactions within condensates at residue-specific resolution [216].

Once homogenous lncRNA is purified, advanced RNA structure probing techniques such as SHAPE-MaP and DMS-MaP can be applied in combination with next-generation sequencing to enable high-throughput structural analysis and accurate determination of secondary structures. These approaches provide a powerful framework for exploring lncRNA structure-function relationships [217]. SHAPE-MaP and DMS-MaP techniques can probe lncRNA flexibility and, when combined with other methods, provide insights into RNA structure within the cellular context by characterizing in vivo nucleotide dynamics [218]. Recently, a method was reported that enables accurate RNA structure probing of all four nucleotides in living cells. By leveraging the unique mutational signature of N1-methylguanine, it improves DMS profiling to capture guanine reactivity, providing richer structural data and supporting more precise RNA structure modeling [219].

As an alternative, a variety of computational techniques, including machine learning, multiple sequence alignment, free-energy estimates, and direct coupling analysis, can be used to predict the secondary structure of RNA [220,221,222,223]. For example, DiffScan, a method for high-resolution analysis of structure probing data, was recently reported to have an improved accuracy in detecting SVRs (structurally variable regions). It can work across multiple platforms like icSHAPE and DMS-seq, showing robust performance in identifying SVRs at nucleotide resolution [224]. Other web server programs such as RNAstructure, Vfold2D, linear-time heuristic, REDfold, and bpRNA-align have also emerged [225,226,227,228,229].

#### 4.2.2. Analysis of LncRNA Tertiary Structure

3D of lncRNAs is very important for understanding their function. LPIs enable the characterization of high-resolution 3D structures of lncRNA. Recent chemical probing-based next-generation sequencing (M2-seq) coupled with mutational profiles has the potential to provide tertiary structural information [230]. Probes like dimethyl sulfate (DMS) and SHAPE (Selective 2′-hydroxyl acylation analyzed by primer extension) react with RNA to reflect structural dynamics. To detect modifications, reverse transcription (RT) or third-generation sequencing methods (e.g., Oxford Nanopore) are commonly used. Techniques like SHAPE-MaP and DMS-MaP-Seq are commonly employed [219,231]. The BASH MaP method has been reported for studying RNA tertiary structure by measuring N7G (N7 position of guanosine) accessibility. When combined with the DAGGER (Deconvolution Assisted by N7G Gaps Enabled by Reduction and Depurination) pipeline, this method reveals alternative RNA conformations, especially for RNAs with G-quadruplexes. This approach has been shown to improve RNA structure prediction, overcoming limitations of traditional methods in analyzing complex RNA structures [232].

In addition, multiple computer algorithms were developed to identify RNA structural ensembles from large data generated by sequencing-based chemical probing, which show increasing potential; examples include DREEM (Detection of RNA Folding Ensembles using Expectation-Maximization), DaVinci (Determination of the Variation in the RNA Structure Conformation through Stochastic Context-Free Grammar), and DANCE-MaP (Deconvolution and Annotation of RiboNucleic Conformational Ensembles Measured by Mutational Profiling) [231,233,234]. Overall, all methods are missing a direct 3D map at the atomic range of the target lncRNA [8].

The tremendous advancement of structural biology technology, particularly cryoEM, has resulted in unparalleled successes in the understanding of proteins and protein complexes that have revolutionized our knowledge of ligand binding, complex formation, and self-assembly—all critical processes in biology [235,236]. Nevertheless, the number of individual lncRNA structural models currently available is limited. This constrains efforts to elucidate their enigmatic structure-function relationships. RNA structural biology has significantly lagged behind protein structural biology [1,237].

To some extent, techniques used for protein 3D characterization can also be applied to lncRNA. X-ray crystallography has succeeded in solving the 3D structures of some RNA [238,239]. Small-angle X-ray scattering experiments can be used to characterize the RNA systems that are too flexible or come in a variety of conformations with low resolution [240]. LncRNAs have also been studied using atomic force microscopy (AFM, Multimode 8, Nanoscope V, Bruker, Santa Barbara, CA, USA), which allows for the analysis of 3D structures in a solution-free environment [241]. In order to differentiate between extended and compact conformations of lncRNA systems, parameters such as 3D size have been evaluated using fluorescence correlation spectroscopy (FCS). For instance, HOTAIR was shown to be less compact than ribosomes but to display a more compact structure when compared to mRNA transcripts [242]. In summary, studies that solely focus on RNA systems have generated a large amount of structural data, but the scope of structure-function roles is still limited by the multiple faces of lncRNA characteristics (e.g., heterogeneity) [12]. Not to mention that characterizing the majority of lncRNA is still challenging due to their intrinsic properties, particularly those containing flexible segments.

##### Single LncRNA–Protein Complex Structural Analysis

**Figure 4:**
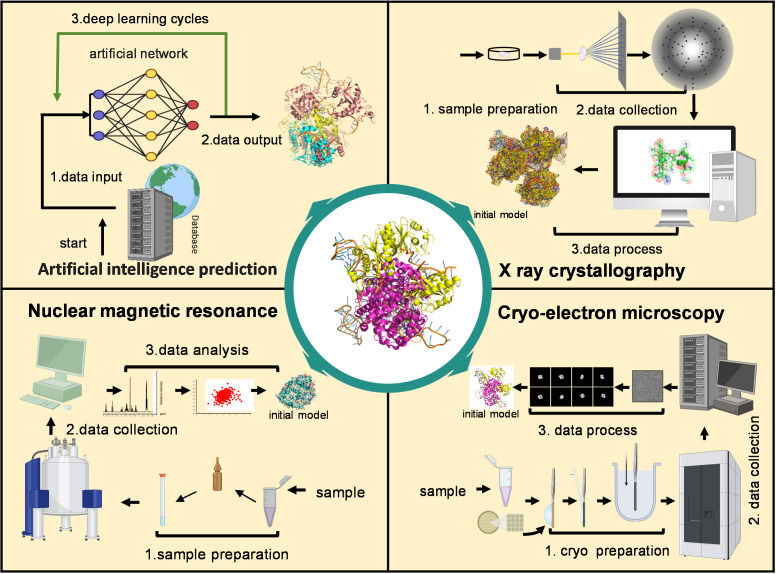
Illustration of current high-resolution 3D structural analysis methods for lncRNA-protein complexes.

Obtaining stable lncRNA samples is the key step for structural observation. Many lncRNAs adopt certain 3D shapes only when induced by binding partners, which are different from linear lncRNAs [240,243]. The protein binding partners help to stabilize lncRNAs for structure determination in these cases. Benefiting from advances in protein structure resolution, structure investigation of lncRNAs can be operationalized by indirectly visualizing the complexes formed by lncRNAs and proteins. Then, in principle, all available approaches used for proteins can be adapted to study lncRNA (Figure 4).

**Figure 5:**
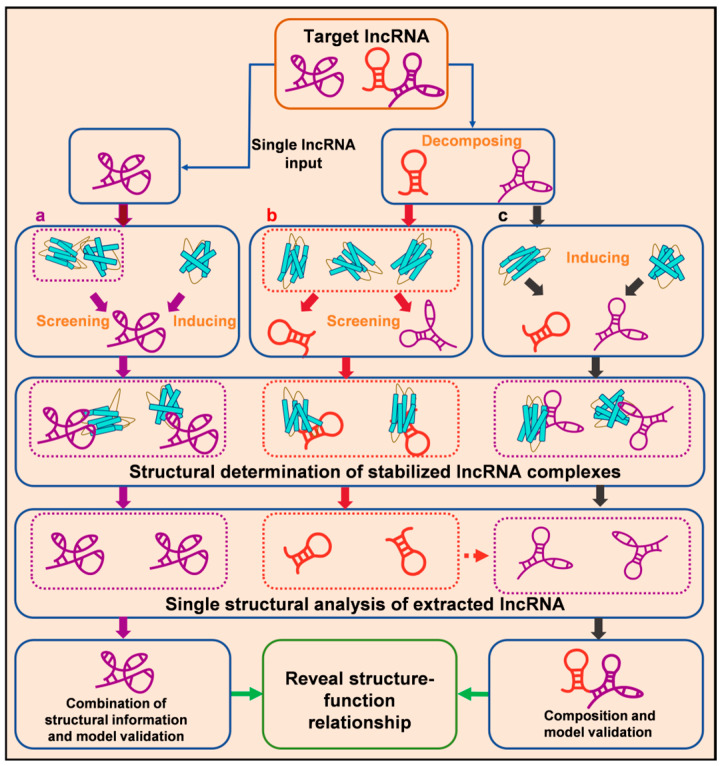
Schematic diagram of proposed integrated structural analysis of lncRNA from the perspective of protein partners; the ISD strategy is incorporated into the pipeline.

Accordingly, we propose a novel strategy to further explore the structural intricacies of lncRNA from the perspective of protein binding, which is ISD (Inducing-Screening-Decomposing). In this strategy, we can firstly induce a protein partner for stabilizing lncRNA. Aditionally, for lncRNA with multiple binding partners, screening for stable partners or parallel analysis is beneficial for comprehensive understanding. Since the lncRNAs harbor a huge range of heterogeneity in size. A significant number of IncRNAs interact with more than one protein; the structure study can be optimized according to functional sub-motifs that regulate the proteins they bind to (decomposing). We have successfully incorporated ISD strategy into our integrated analysis pipeline (refer to Figure 5). This is especially important when it comes to the functional characteristics of lncRNA, because low abundance and multiple functional roles in cells require lncRNA to interact stoichiometrically with target molecules so as to have detectable regulatory effects [30].

With regard to available techniques, recent advances in artificial intelligence (AI) demonstrate great potential in structural prediction [244,245]. Tools like AlphaFold and ProteinBERT (RFAA) have demonstrated remarkable accuracy in predicting protein structures [246,247]. Their accuracy decreases when predicting the binding mechanisms of complexes, especially at the interface [248,249]. An effective compensatory approach is to use proteins with known structures to predict unknown lncRNA partners. Nevertheless, AI-driven deep learning algorithms are rapidly evolving and continuously improving their accuracy on protein complexes. For example, RoseTTAFoldNA has been shown to efficiently generate 3D structural models for protein-RNA complexes with higher accuracy [250]. Other deep-learning-based methods include DeepDISOBind and AI-integrated networks, while MSAs (multiple sequence alignments) provide essential input data for these tools [251,252,253].

Overall, lncRNA structure prediction can be approached in multiple steps, and integrating protein-binding information can significantly enhance prediction accuracy. For example, first identifying protein interaction partners (e.g., via CLIP-seq data), then predicting secondary structure with RNA-binding constraints using tools like SPOT-RNA, followed by protein-guided tertiary modeling through integrative methods such as RoseTTAFoldNA or constrained docking, and finally validating models with geometric checks and simulations. For instance, AlphaFold3 has been released with the aim of extending accurate predictions to RNA structures and RNA–protein complexes. However, recent evaluations suggest that AlphaFold3 currently struggles with non-canonical RNA interactions, and its predictions in this domain remain unreliable, especially where training data are scarce. It is therefore recommended to combine AI-based predictions with docking or structure-informed approaches for now [254]. Table 2 summarizes common AI-based methods for modeling lncRNA structures and their complexes with proteins.

In addition, databases such as MARS (Master database of All possible RNA Sequences) and ViRBase v3.0 greatly expand the scope of RNA homology search by integrating multiple large-scale RNA and genome resources, thereby improving alignment quality and structure prediction accuracy [255,256].

**Table 2:** Common AI-based methods for modeling lncRNA structures and their complexes.

Tool Name	RNA Struct.	lPI Struct.	Key Features	Suitability	Ref.
RNA-Composer	Yes	No	Fast conversion from 2D to 3D structure	Suitable for short lncRNA fragments with known secondary structure, web-based and fast	[257]
3dRNA v2.0	Yes	Limited	Assembles 3D structure from motifs and 2D input	Suitable for moderate-length lncRNAs, accuracy depends on quality of 2D input	[258]
P-FARFAR2	Yes	Limited	Parallelized enhancement of FARFAR2, multithreaded greedy sampling for low-energy structure assembly	Suitable for high-precision RNA 3D modeling, better efficiency and accuracy over FARFAR2, especially for complex/larger RNAs	[259]
FARNA/FARFAR2	Yes	Limited	High-precision folding, but computationally intensive	Best for small lncRNA segments, high-quality modeling with heavy compute requirements	[260]
RNA-MSM	Indirectly	No	MSA-based RNA language model, captures structural information via attention maps and embeddings	Outperforms BERT-like RNA models in predicting base-pairing and solvent accessibility, suitable for structure-function tasks	[261]
MoEFold2D	No (2D)	No	Mixture-of-experts model combining DL and physics-based predictions, auto-detects in/out-of-distribution sequences	Offers high accuracy for in-distribution and robust predictions, useful when training/test domains differ	[262]
RNA-LLM Folding	No	No	Evaluates multiple pretrained RNA language models for RNA secondary structure prediction	Provides benchmark datasets and unified experimental framework, shows LLMs can improve prediction in high-homology regions, with limitations in generalization	[263]
RPI-SE	No	Yes	Ensemble model using PWM, Legendre moments (proteins), and k-mer features (ncRNAs), high accuracy and robustness	Effective computational predictor for ncRNA-protein interactions, suitable for accelerating interaction studies with sequence data only	[264]
SPOT-RNA2	Yes	No	High-accuracy secondary structure prediction, supports pseudoknots	Great for generating input structures for 3D modeling of lncRNA	[261]
MXfold2	No	No	Combines neural networks and thermodynamics for better generalization	Fast and accurate, ideal for batch secondary structure prediction	[265]
RNAstructure	No	No	Supports SHAPE data and pseudoknots, energy minimization	Stable and widely used, excellent for combining with experimental data	[266]
IntaRNA	No	Yes	Predicts RNA–protein interaction regions	Useful for predicting lncRNA binding regions with proteins	[267]
catRAPID	No	Yes	Sequence-based scoring of RNA–protein interaction strength	Useful for large-scale screening of LPIs	[196]
RPI-EDLCN	No	Yes	Ensemble deep learning framework based on CapsuleNet, integrates sequence, secondary structure, motif, and physicochemical features, used for feature extraction	High accuracy in predicting ncRNA-protein interactions across multiple datasets, effective across species	[268]
LPI-MFF	No	Yes	Multi-source information fusion (PPI, sequence, structure, physico-chemical), feature selection by random forest	High accuracy and robustness across multiple datasets, good generalization for RPI prediction	[269]
DRPScore	Yes	Yes	Deep learning model for identifying native-like RNA-protein complex structures across docking types (bound/unbound)	Suitable for RNA-protein complex modeling, high accuracy in selecting native-like structures even under flexible docking scenarios	[25]
AlphaFold3	Yes	Yes	Accurate protein 3D structure prediction, limited in RNA/RNA-protein complex	Provides protein structure for docking; essential if structure is unknown	[254]
RoseTTAFold	Yes	Yes	Deep learning-based 3D structure prediction for RNA-protein complexes, with confidence scores	Suitable for predicting structures of complexes without known homology	[250]

Experimental methods play a crucial role in validating these predicted structural models. X-ray crystallography has long provided high-resolution insights into protein-RNA complexes, providing high-resolution structures that reveal detailed molecular interactions once the target lncRNA is stabilized. For instance, TRIM71 interacts with the lncRNA Trincr1 to repress the FGF/ERK pathway in mouse ESCs (embryonic stem cells). Recently an X-ray crystallography study determined an RNA motif in Trincr1 that is specifically recognized by TRIM71 [270]. Nuclear magnetic resonance (NMR) imaging has been extensively used to study small-scale systems. It provides a precise way to record details about RNA dynamics, its various configurations, and the speeds at which these configurations change [271]. Fragmented (decomposing) regions or motifs of lncRNA with a stabilized protein can be employed in an NMR pipeline [240,241].

With recent advances in cryo-electron microscopy (cryoEM), the number of RNA and protein-RNA structure models in the PDB has grown significantly. For instance, the non-coding 7S RNA inhibits transcription via mitochondrial RNA polymerase dimerization. The dimerization of POLRMT is induced by interactions with 7S RNA. CryoEM analysis, focusing on 2D classification and 3D reconstruction, obtained a low-resolution structure of POLRMT-POLRMT complexes. By using decomposition and negative control methods, researchers were able to map the density of 7S RNA, which appears to bind and regulate the wrapping of POLRMT during dimerization [272]. In the study of lncRNA ADIPINT interacting with pyruvate carboxylase in adipocyte metabolism, the RNA was sequentially truncated to explore its binding patterns with proteins, using methods analogous to the decomposition techniques proposed in our ISD proposal [273].

##### Integrated Structural Approaches

It is essential to select and optimize approaches tailored to each target’s characteristics. While a single method may not fully capture lncRNA dynamics, integrated structural analysis can overcome these limitations and enhance the accuracy of structural determination [274,275]. As Figure 5 shows, we propose an overall diagram to reveal the structure-function relationship of lncRNA. ISD strategies are expected to be used during implementation.

A typical example is the ribosome, which has been examined using various technologies over time. Generally, small-angle X-ray scattering (SAXS) is generally used as an initial approach for higher-resolution structure determination, paving the way for techniques like X-ray crystallography or cryo-electron microscopy. The sample preparation requirements for SAXS are relatively less stringent. A low-resolution structure obtained from SAXS is a valuable resource for further high-resolution modeling and complex studies in cryo-EM. For example, SAXS was utilized to determine the low-resolution structures of the Braveheart lncRNA and the Braveheart-CNBP ribonucleoprotein complex. These results were supported by computational 3D modeling and phylogenetic analysis [240].

## 5. Development of Novel Biomedical Interventions Targeting LPIs

Emerging technologies are being developed to target lncRNA-protein for disease treatment, given its essential roles in biological processes. Traditionally, methods such as small interfering RNAs (siRNAs) and antisense oligonucleotides (ASOs) have been used to degrade lncRNA by directly targeting through base pairing. A notable concern for these methods is the off-target effects [276,277,278]. Advanced approaches, such as CRISPR/Cas9, can be applied for lncRNA knockout regardless of cellular location but are limited by a relatively narrow spectrum of targetable lncRNAs [279,280].

Since LPIs play a vital role in diverse diseases, as discussed above, specifically focusing on the interactions provides creative solutions for broadening the range of targetable LncRNA in drug development. Two main approaches are currently available: The first approach involves the development of small molecules that target lncRNA-bound protein partners to intervene in lncRNA-associated physiological processes. In fact, protein-based drug design occupies a very important position in pharmaceutical areas [281] due to factors such as the close connection between protein structure and function, the abundance of atomic models obtained by state-of-art technologies, and the potential for compound optimization [282,283,284]. For example, Lin28 is overexpressed in many cancer cells and disrupts the maturation of RNA let-7 by directly binding to let-7 microRNA precursors. Small-molecule Lin28 inhibitors have been discovered to prevent Lin28 from binding to let-7, presenting a promising therapeutic strategy [285]. Numerous Lin28 inhibitors have been identified through various high-throughput screening (HTS) techniques [286,287].

The second approach focuses on targeting the interaction interface by designing molecules that disrupt the spatial structure of lncRNAs or interfere with LPIs. For instance, even though EZH2 (Enhancer of zeste homolog 2) is an attractive drug target for cancers, the design of EZH2 inhibitors is constrained by diverse undesigned effects on gene expression. The lncRNA BDNF-AS (brain-derived neurotrophic factor antisense) binds EZH2 in a concentration-dependent manner. AlphaScreen was employed to quantify lncRNA-EZH2 interaction, leading to the identification of a small molecule, ellipticine, which effectively inhibits the interaction between BDNF-AS and EZH2 [288,289]. This study highlights the effectiveness of high-throughput screening in identifying regulators of LPIs. Furthermore, it has been reported that the ENE (element for nuclear expression) triplexes in lncRNA MALAT1 and KSHV PAN serve as unique platforms for protein binding, such as MALAT1 and KSHV PAN, and serve as unique platforms for protein binding, including interactions with methyltransferase-like protein 16 (METTL16). Several small ligands have been discovered that selectively bind to the ENE triplexes and disrupt these protein-lncRNA interactions [290,291].

## 6. Conclusions

As more lncRNAs have been discovered in recent years, their roles in cellular activities are receiving growing attention. Understanding the cellular mechanism of lncRNA, particularly for those with advanced structures, is crucial for studying the significant correlation between structure and function. However, due to the distinguishable properties compared to proteins, it is difficult to explore the structure-function relationship of lncRNA solely from resolving certain configurations formed by their sequences. Moreover, the availability of high-resolution structures for individual lncRNAs remains extremely limited. Additionally, predicting the structure or function of lncRNAs through sequence alignment is largely constrained by the low conservation of their sequences.

We emphasize the importance of focusing on the LPI in lncRNA research, as proteins have significantly contributed to a more comprehensive understanding of lncRNA in various contexts. By expanding attention from lncRNA itself to its interaction with the primary protein ligand, LPIs can be treated as functional units. This approach, on the one hand, enables deeper insights into the cellular roles of lncRNAs from a broader biological process perspective. On the other hand, utilizing proteins as stabilizing ligands for lncRNAs during structural investigations can effectively address many of the challenges faced in lncRNA research, as discussed in the main section.

Protein binding is a critical step for lncRNAs to exert their functions. Structural information obtained from solving lncRNA-protein complexes provides a robust foundation for interpreting the structure-function relationships of lncRNAs, which could subsequently facilitate the development of novel therapeutic strategies. Except for structure-based small compound screening from bond protein or lPIs. Strategies such as antisense oligonucleotides and siRNAs can all be designed to target functional domains or disrupt essential structural regions of lncRNAs, thereby selectively interfering with their pathological roles. While notable progress has been made, the clinical application of lncRNA-targeted treatments still faces challenges. These include poor sequence conservation across species, extensive alternative splicing, and tissue-specific localization, all of which complicate the development of reliable disease models and functional studies. Off-target effects and delivery specificity also present ongoing concerns, particularly for systemically administered therapeutics. Integrative strategies such as transcriptome-wide association studies (TWAS), along with improved disease models and structure-based screening, will be essential to overcome these barriers and facilitate the development of safe, effective, and precise lncRNA-based interventions. In this review, we have examined the physiological roles of lncRNAs and provided a comprehensive overview of recent advances in LPIs. We further summarized progress in lncRNA-associated diseases and structure-based drug design centered on LPIs, aiming to inspire further developments of innovative treatments in the future.

## Figures and Tables

**Table 1:** Illustrates commonly used methods for detecting LPIs.

	Method	Principle	Advantages	Limitations	Ref.
Protein-Centric	RIP-Seq/RIP-chip	Immunoprecipitation of RBP, followed by RNA identification	Simple, applicable in vivo	Cannot map precise binding sites	[155]
	CLIP-Seq (HITS-CLIP, iCLIP, PAR-CLIP)	UV crosslinking, IP of RBP, and sequencing	Single-nucleotide resolution; transcriptome-wide	Labor-intensive,RNA loss during purification	[156,168,191]
	eCLIP	Enhanced CLIP with size-matched input control	Improved specificity and reproducibility	Requires large sample amounts	[160]
	fCLIP	Formaldehyde-based crosslinking CLIP variant	Captures transient or weak interactions	Lower resolution compared to UV-based CLIP	[161]
	RBNS	In vitro pull-down of lncRNAs with immobilized RBP	Quantifies binding affinity, statistical modeling	In vitro only, prone to false positives	[152]
	seCLIP	Size-matched input and simplified protocol to purify and sequence LPIs	High resolution, efficiency, and scalability	Still relies on UV crosslinking and may require optimization	[192]
RNA-Centric	IVT RNA	Biotin-labeled RNA baits used to isolate RBPs	Straightforward, customizable	In vitro only, RNA misfolding risks	[165] [193]
	CHIRP/CHART/RAP	Hybridization of biotinylated probes to native RNA, IP of LPIs	In vivo relevance, genome-scale coverage	Requires high expression levels and probe optimization	[170]
	MORPH-MS	Multiplex probe hybridization followed by MS	Efficient RNA retrieval with fewer oligos	Still dependent on transcript abundance	[173]
	RAP-MS	Biotinylated antisense probes capture target RNA and associated proteins, proteins identified by MS	Specific, enables in vivo RBP identification	Requires effective probe design, low resolution for binding sites	[172]
	RPL (RNA Proximity Labeling)	Labeling enzymes are directed to specific RNAs to biotinylate nearby proteins	In vivo compatible, identifies weak/transient interactions	Requires expression optimization	[174]
	CARPID	CRISPR assisted RNA–protein interaction detection	Specific to endogenous RNAs, works without crosslinking	Limited by gRNA design and accessibility	[175]
In Silico	LPICGAE, DFRPI, DeepLPI	Machine learning-based prediction from sequence and structural data	Fast screening, captures nonlinear patterns	Dependent on training data quality	[176,179,194]
	RNAInter, RPISeq, catRAPID	Rule-based or probabilistic sequence feature models	Web-accessible, user-friendly	Limited performance on unseen RNA–protein pairs	[67,195,196]
	RNAincoder, SoCube, DeepBtoD	Advanced neural nets trained on interaction databases	Improved performance for large-scale predictions	Limited for low-abundance RNAs	[185,186,188]
	DeepA-RBPBS	Deep learning model using CNN, BiGRU, and attention to predict RBP binding sites from RNA sequence and structure	Captures both sequence and structural features, effective across multiple RBPs	Relies on well-annotated training data, may be sensitive to feature encoding quality	[189]
	PRIESSTESS	Using regression on binding data, integrating both sequence and secondary structure features	Captures both sequence and structure specificity, applicable to diverse RBPs	May oversimplify complex binding rules, depends on quality of input data	[197]
	RBPNet	Predicts CLIP-seq crosslink count distribution from RNA sequence at single-nucleotide resolution, using bias correction and attribution methods	High-resolution predictions, supports eCLIP, iCLIP, and miCLIP, captures known and novel motifs, enables variant impact analysis via in silico mutagenesis	Requires large-scale training data, computationally intensive, performance depends on data quality and assay consistency	[198]
	DeepFusion	Integrating RNA sequence and in vivo structural features from DMS-seq to predict RBP binding	Combines local motifs and global context, improved accuracy over sequence-only models, applicable to RNA degradation studies	Depends on high-quality DMS-seq data, computationally intensive	[199]
	HDRNet	Deep learning-based framework to predict dynamic RBP binding events under diverse cellular conditions	High accuracy in cross cell type prediction, interpretable motifs, applicable to disease association studies	Requires large, diverse annotated datasets, black-box model may limit mechanistic insight	[200]
	qNABpredict	Predicts nucleic acid-binding residue content using support vector regression and computed features from protein sequence	Much faster than residue-level methods, provides informative summary, suitable for large scale analysis	Less detailed than residue-level tool, focuses on content rather than precise binding residues	[201]
	HybridRNAbind	Meta-model integrating structure- and disorder-trained predictors for RNA-binding residues	Reduces cross-predictions, works well across structured and disordered regions	Performance depends on quality/diversity of input models, modestly effective on non-matching annotations	[202]
	pyRBDome	Pipeline integrating ML-based RBS predictions with structural data to refine RBPs datasets	Enhances confidence in RNA-binding proteome data, improves RBS detection via ensemble learning	Relies on existing structure/ML data quality, structural benchmarks still have known limitations	[203]

## Data Availability

Not applicable.
